# Parasporin-2 from a New *Bacillus thuringiensis* 4R2 Strain Induces Caspases Activation and Apoptosis in Human Cancer Cells

**DOI:** 10.1371/journal.pone.0135106

**Published:** 2015-08-11

**Authors:** Kevin Brasseur, Pascal Auger, Eric Asselin, Sophie Parent, Jean-Charles Côté, Marc Sirois

**Affiliations:** 1 Research Group in Molecular Oncology and Endocrinology, Department of Medical Biology, Université du Québec à Trois-Rivières, 3351, Boul. Des Forges, CP 500, Trois-Rivières, Québec, Canada G9A 5H7; 2 Agriculture and Agri-Food Canada, Research Centre, 430, Boul. Gouin, Saint-Jean-sur-Richelieu, Québec, Canada J3B 3E6; University of Pecs Medical School, HUNGARY

## Abstract

In previous studies, parasporin-2Aa1, originally isolated from *Bacillus thuringiensis* strain A1547, was shown to be cytotoxic against specific human cancer cells but the mechanisms of action were not studied. In the present study, we found that proteinase K activated parasporin-2Aa1 protein isolated from a novel *B*. *thuringiensis* strain, 4R2, was specifically cytotoxic to endometrial, colon, liver, cervix, breast and prostate cancer. It showed no toxicity against normal cells. Upon treatment with proteinase K-activated parasporin-2Aa1, morphological changes were observed and western blot analysis revealed the cleavage of poly (ADP-Ribose) polymerase, caspase-3 and caspase-9 in cancer cell lines exclusively, indicative of programmed cell death, apoptosis. Flow cytometry analyses,using propidium iodide and annexin V, as well as a caspases 3/7 assay confirmed apoptosis induction. Further analyses were performed to study survival pathways, including AKT, XIAP, ERK1/2 and PAR-4, a known inducer of apoptosis. These results indicate that parasporin-2Aa1 is a selective cytotoxic protein that induces apoptosis in various human cancer cell lines from diverse tissues.

## Introduction

*Bacillus thuringiensis* is a Gram-positive bacterium that produces crystalline parasporal inclusions during sporulation. These inclusions are made of proteins, the δ-endotoxins. They are classified into two families, the crystal (Cry) and the cytolytic (Cyt) proteins encoded by the *cry* and *sit* genes, respectively [1,2]. The Cry proteins have been extensively studied since 1970’s owing to their specific insecticidal activities against lepidoptera, dipteran and coleopteran [3]. Upon ingestion by a susceptible insect, the parasporal inclusions are solubilized in the alkaline insect midgut, the Cry protoxins are released and then processed by midgut proteases to yield activated toxin proteins. These bind to specific receptors located on the membrane of epithelial gut cells, leading to pore formation and ultimately to insect death [1,4].

The successful development and use of *B*. *thuringiensis*-based formulations for the control of insect pests has led to the isolation of thousands of novel strains and hundreds of Cry proteins have now been characterized to various extents [5]. Several studies have shown that non insecticidal Cry proteins are more widely distributed than the insecticidal Cry proteins [6]. This led to the research of potentially novel biological activities from the non-insecticidal Cry proteins. Following a large screening, some non-insecticidal and non-hemolytic Cry proteins showed cytotoxic activity against human cancer cells and these new *B*. *thuringiensis* toxins were called parasporins [7,8]. So far, six families of parasporins, PS1 –PS6, have been identified [9]. Each parasporin family exhibits specific spectrum and mechanism of action against human cancer cells.

Parasporin-2Aa1 (PS2Aa1, also classified Cry46Aa1) produced by *B*. *thuringiensis* serovar *dakota* strain A1547 has been intensively investigated for its toxic action in cancer cells [9–11]. When activated by proteinase K, PS2Aa1 is at least 400- fold more toxic for the human cancer cell line HepG2 (human hepatocyte cancer) than for the normal human cell line HC (human normal hepatocyte) and human cancer cell line HeLa (human uterine cervical cancer) [12]. In HepG2 cells, the monomeric toxin appears to bind to an unknown receptor protein located in the lipid raft [13]. Once linked to the receptor, PS2Aa1 oligomerizes to permeabilize the membrane leading to pore formation [11,12]. A Glycosylphosphatidylinositol (GPI)-anchored protein appears to be involved for the efficient cytocidal action of PS2Aa1 [13]. Pore formation results in alterations of the cytoskeletal structures, fragmentation of organelles, alterations of cell morphology such as cell swelling and finally cell lysis [11]. The mode of cell death appears to be non-apoptotic but this hypothesis was not confirmed [11–13]. Thus, additional characterisation of the intracellular events involved during induced- PS2Aa1 cell death was mandatory to confirm whether or not apoptosis was involved.

In this present study, an additional *B*. *thuringiensis* strain called *Bt* 4R2 which contain the gene encoding the Cry46Aa1 protein (PS2Aa1) has been studied to identify the mechanisms involved in cytocidal-dependent cell death induction. We found that PS2Aa1 was very cytotoxic to many cancer cells *in vitro*. To further explore the mechanism using selected cancer cells from different tissue (HepG2-hepatocyte cancer, PC-3-prostate cancer and MCF-7-breast cancer) we found that apoptosis cell death was occurring via caspases and poly (ADP-ribose) polymerase (PARP) cleavage. We also found that PS2Aa1 shows very low toxicity to normal cell lines (IOSE-144, HIESC, HIEEC and MCF-10A).

We further support the hypothesis of apoptosis induction with identification of various survival pathway inhibition including AKT, XIAP, ERK1/2 and induction of the tumor suppressor PAR-4 following treatment with PS2Aa1. We also found out that inhibiting the Pi3K/AKT pathway in combination with the toxin increases, in a synergetic manner, the efficiency of PS2Aa1 to induce apoptosis in cancer cells. Thus, PS2Aa1 appears to be a cell-killing discriminating toxin regulating apoptosis in different human cancer cells.

## Materials and Methods

### Bacterial strain and culture media

*B*. *thuringiensis* serovar *dakota* strain 4R2 was used in this study. It was obtained from the *Bacillus* Genetic Stock Center (Ohio State University, Columbus, OH, USA). Bacterial cells were grown at 30°C on nutrient agar from Sigma-Aldrich (St-Louis, MO, USA) at pH 7.1.

### Cells and culture conditions

Human hepatocyte cancer cell line HepG2 (HB-8065), human prostate cancer cell line PC-3 (CRL-1435), human epithelial colorectal adenocarcinoma cell line Caco-2 (HTB-37), human epithelial cervix adenocarcinoma cell line HeLa (CCL-2), human uterus endometrium adenocarcinoma cell line Hec-1A (HTB-112), human uterus endometrium adenocarcinoma cell line KLE (CRL-1622), human breast adenocarcinoma cell line MDA-MB231(HTB-26), human breast cancer cell line MCF-7 (HTB-22), human non-tumorigenic epithelial cells MCF-10A (CRL-10317), human epithelial ovary adenocarcinoma cell line OVCAR-3 (HTB-161) and human epithelial ovary adenocarcinoma cell line SKOV-3 (HTB-77) were obtained from the American Type Culture Collection (ATCC). Human immortal non-tumorigenic ovarian surface epithelial cell line IOSE-144 was kindly provided by Dr. David Hunstman (British Columbia Cancer Research Center, Vancouver, BC, Canada). Human immortal endometrial stromal cells HIESC and Human immortal endometrial epithelial cells HIEEC were a kind gift and produced by Dr. Michel Fortier (Centre Hospitalier de l’Université Laval, Quebec City, QC, Canada) [14]. Human ovarian carcinoma cells A2780 were kindly provided by Dr. G. Peter Raaphorst (Ottawa Regional Cancer Center, Ottawa, ON, Canada). Human endometrial adenocarcinoma cell line Ishikawa was kindly provided by Dr. Samuel Chogran (Université de Montréal, Montreal, QC, Canada). HepG2, PC-3, HIEEC and HIESC cells lines were maintained in RPMI 1640 medium containing 10% foetal bovine serum and 50 μg/ml gentamycin. MCF-7 and OVCAR-3 cell lines were maintained in RPMI 1640 medium containing 10% bovine growth serum and 50 μg/ml gentamycin. MDA-MB-231 cell line was maintained in RPMI 1640 medium containing 5% bovine growth serum and 50 μg/ml gentamycin. Hec-1A cell line was maintained in McCoy’s medium containing 5% bovine growth serum and 50 μg/ml gentamycin. SKOV-3 cell line was maintained in McCoy’s medium containing 10% bovine growth serum and 50 μg/ml gentamycin. HeLa, Ishikawa and A2780 cell lines were maintained in DMEM-F12 medium containing 2% bovine growth serum and 50 μg/ml gentamycin. MCF-10A cell line was maintained in DMEM-F12 medium containing 5% foetal growth serum, 20 ng/ml EGF, 0.5 mg/ml hydrocortisone, 100 ng/ml cholera toxin, 10 μg/ml insulin and 1X Penicillin-Streptomycin. KLE cell line was maintained in DMEM-F12 medium without HEPES containing 10% bovine growth serum and 50 μg/ml gentamycin. Caco-2 cell line was maintained in DMEM-F12 medium containing 10% foetal bovine serum and 50 μg/ml gentamycin. IOSE-144 cell line was maintained in MCDB105 medium containing 10% foetal bovine serum and 50 μg/ml gentamycin. All cells were maintained at 37°C with 5% CO_2_.

### Total DNA isolation

Total DNA from *B*. *thuringiensis* 4R2 was isolated from a 5mL overnight culture using QIAmp DNA blood mini kit (Qiagen, Toronto, ON, Canada), according to the manufacturer’s instructions for bacterial DNA extraction.

### PCR amplification

The primers used in this study were designed in our laboratory from the *cry*46Aa1 gene nucleotide sequence. Primers for PCR amplification were as follows: *Bt*4R2-2F: 5’- TAACCGGAGGGCTTCAAG -3’ (sense) and *Bt*4R2-1R: 5’- TAATTCCCCCATTTTGGG -3’ (antisense). PCR were conducted in an Applied Biosystems 2720 thermal cycler (Life Technologies, Ottawa, ON, Canada). The PCR reactions were performed in a 50μL volume containing 200μM each of deoxynucleoside triphosphates (dNTPs), 1X PCR buffer, 0,5μM of each primers, 100ng of DNA and 1,25 units of Taq DNA polymerase. All PCR reagents were from New England Biolabs (Ottawa, ON, Canada). PCR was performed by an initial denaturation step at 94°C for 5 min, followed by 30 cycles at 94°C for 45 s, 50°C for 30 s, 72°C for 2 min and a final extension at 72°C for 7 min. PCR products were size separated on a 1% agarose gel and visualized using SYBR-Safe (Invitrogen, Burlington, ON, Canada) staining upon UV transillumination.

### DNA sequencing

Amplicons were purified using the Mini Elute PCR Purification Kit from Qiagen. The purified PCR reactions were resolved on a 3130XL Genetics Analyser (Applied Biosystems) at IBIS Plate-forme d’Analyses Génomiques (PAG) de l'Université Laval (Quebec City, QC, Canada). Both strands of the DNA sequence were sequenced: The 940pb sequence was compared using the Blast-N tool (National Center for Biotechnology Information; www.ncbi.nim.nih.gov).

### Preparation of activated parasporal proteins

*Bacillus thuringiensis* strain 4R2 was cultivated on nutrient agar plates, incubated for 4 days at 30°C until cell lysis. The cells were harvested from the plates and washed twice with sterile distilled water. The pellet containing the spores-crystal proteins- was solubilized in 500μL of solubilisation buffer containing 56mM Na_2_CO_3_ (pH:11,4) and 11mM dithiothreitol (DTT) for 1 h at 37°C. Insoluble material was pelleted by centrifugation at 13 200 rpm for 2 minutes and the supernatant was passed through a 0, 22μm membrane filter. 250μL of the filtrate was transferred to a sterile 1,5mL centrifuge tube and the pH adjusted to 8 with 1M Tris-HCl (pH 4,98).

The solubilised proteins were digested with either proteinase K (final concentration at 185μg/mL) or trypsin (final concentration at 300μg/mL) for 1 h at 37°C. Phenylmethylsulfonyl fluoride (PMSF) was added (final concentration 1 mM) to stop proteolytic processing. To confirm the presence of the parasporal proteins, SDS-PAGE analysis was performed as described elsewhere [15] using 4% stacking gel and 12% separating gel. After electrophoresis, the gel was stained with 0, 1% Coomassie blue R-250 (Sigma-Aldrich). Protein concentration was determined with the Bio-Rad DC Protein Assay (Bio-Rad Laboratories, Mississauga, ON, Canada).

### Assay of cytotoxicity

The antiproliferative activity of *B*. *thuringiensis* 4R2 toxin proteins was assessed using the MTT assay [16,17]. Briefly, plates were seeded with 180μL of normal and cancer cells in suspension (for HIESC, 14 000; IOSE-144, 12 000; HIEEC, 15 000; Ishikawa, 16 000; HeLa, 16 000; KLE, 14 000; Hec-1A, 12 000; Caco-2, 20 000; PC-3, 16 000; HepG2, 20 000; A2780, 16 000; OVCAR-3, 20 000; SKOV-3, 14 000; MCF-7, 16 000; MDA-MB231, 12 000 and MCF-10A, 8000) in medium using 96-wells plates. Plates were incubated at 37°C, 5% CO_2_ for 24h. Freshly solubilized and activated *B*. *thuringiensis* 4R2 toxin proteins (with proteinase K or trypsin) in solubilisation buffer were diluted in fresh medium, and 100μL aliquots containing escalating concentrations of the toxin proteins (0μg/mL to 20μg/mL) were added and the plates were incubated for another 24h. The final concentration of the solubilisation buffer (56mM Na2CO3, 11mM DTT, 100μg/mL proteinase K (or 30mg/mL trypsin) and 1mM PMSF) in the culture media was 8% and was kept constant in all experiment. After 24h, 10μL of 3-(4,5-dimethylthiazol-2-yl)-2,5-diphenyltetrazolium bromide (MTT) (5mg/mL in PBS) were added to the wells. Four hours later, 100μL of the solubilisation solution (10% sodium dodecyl sulfate (SDS) in 0,01 M HCl) were added and the plates incubated overnight (37°C, 5% CO_2_). The optical density was read using a Fluostar optima BMG (BMG Labtech Inc., Durham, NC, USA) at 565nm. Readings obtained from treated cells were compared with measurements from control cell plates fixed on the treatment day; and the percentage of cell growth inhibition was calculated for each toxin protein (proteinase K activated or trypsin activated). The experiments were performed in triplicate. The assays were considered valid when the coefficient of variation for a given set of conditions and within the same experiment was < 10%.

### Light microscopy observation

For observation of the morphological changes, normal (HIESC) and cancer cells (PC-3, MCF-7 and HepG2) were observed after 24h treatment with proteinase K activated toxin proteins at 1μg/mL (MCF-7) or 2μg/mL (HIESC, PC-3 and HepG2). 10X and 20X magnifications were used with an Olympus (model BX60) light microscope (Carsen Group, Markham, ON, Canada).

### Antibodies and reagents

The Na_2_CO_3_, DTT, 3-(4,5-dimethylthiazol-2-yl)-2,5-diphenyltetrazolium bromide, HCl, SDS, PMSF, Tris-HCl and proteinase K were all obtained from Sigma-Aldrich. All primary antibodies were obtained from Cell Signaling Technology (Bervely, MA, USA) except for β-actin (Sigma-Aldrich). Secondary antibody, HRP-conjugated goat anti-rabbit were purchase from Bio-Rad Laboratories. Annexin V/PI apoptosis kit was purchased from Invitrogen. MEK ½ inhibitor, U0126, was obtained from Cell Signaling Technology and PI3K inhibitor, Wortmannin, was obtained from Sigma-Aldrich.

### Western blot

PS2Aa1 treated cells were washed with PBS and submitted to lysis in cold RIPA buffer containing protease inhibitors (Complete from Roche Applied Science, Laval, QC, Canada) and phosphatase inhibitor (PhosSTOP from Roche Applied Science) followed by three freeze-thaw cycles. Equal amounts of cell lysates, determined using Bio-Rad DC protein assay, were separated on polyacrylamide gels (10–14%) and transferred on nitrocellulose membranes (Bio-Rad). Membranes were blocked in 5% milk, PBS 1X, 0.06% Tween 20 for 1 h at room temperature, probed with primary antibody, washed in PBS 1X, 0.06% Tween 20, and incubated with horseradish peroxidase-conjugated secondary antibody (Bio-Rad). Detection was performed using SuperSignal West Femto substrate (Thermo Fisher Scientific, Nepean, ON, Canada), as described by the manufacturer using UVP bioimaging systems.

### Measurement of Annexin V/PI cells

FITC Annexin V/Dead Cell Apoptosis Kit (Molecular Probes Inc., Eugene, Oregon, USA) was used according to the manufacturer’s instructions. Briefly, the treated cells were collected, washed with PBS and diluted in 1X Annexin Binding buffer (100 μl). For each sample, 5 μL of Annexin V and 2 μl of Propidium Iodide (PI) were added to the cell suspension and incubated for 15min at room temperature. An additional 100 μl volume of Annexin binding buffer was added to each sample for a total of 200 μl. Samples were analyzed (10 000 events) using a Beckman Coulter flow cytometer Cytomics FC500. Analyses were performed using CXP Analysis software (Beckman Coulter, Mississauga, ON, Canada).

### Caspase 3–7 assay

To measure the specific activity of caspase 3 and 7, a luminescent assay kit named Caspase-Glo 3/7 assay (Promega, Madison, WI, USA)) was used. This assay provides a proluminescent caspase-3/7 substrate containing a sequence (DEVD) specific to caspase 3 and 7. If caspases 3 and/or 7 are active, the substrate is cleaved and aminoluciferin will be emitted. Briefly, plates were seeded with 180μL of normal and cancer cells in suspension (for HIESC, 14 000; PC-3, 16 000; HepG2, 20 000 and MCF-7, 16 000) in medium. Plates were incubated at 37°C, 5% CO_2_ for 24h. Freshly solubilized and activated *B*. *thuringiensis* 4R2 toxin proteins (with proteinase K) in solubilisation buffer were diluted in fresh medium. Then, 100μL aliquots containing the toxin, with or without inhibitors, were added and the plates were incubated for another 24h. The final concentration of the solubilisation buffer (56mM Na2CO3, 11mM DTT, 100μg/mL proteinase K and 1mM PMSF) in the culture media was 8% and was kept constant in all experiment. After 24h, 100μL of Caspase-Glo 3/7 reagent was added to the wells. One hour later, the optical density was read using a Fluostar optima BMG (BMG Labtech Inc., Durham, NC, USA). Readings obtained from treated cells were compared with measurements from control cells using fold increases. Each experiments were performed in duplicate.

### Statistical analyses

The data were subjected to either one-way analysis of variance or Student’s t-test (PRISM software version 5.00; GraphPad, San Diego, CA). Differences between experimental groups,when using one-way analysis of variance, were determined by the Tukey's test. Statistical significance was accepted when p < 0.05.

## Results

### Characterization of B. thuringiensis 4R2 cytotoxic crystal protein

**Fig 1:**
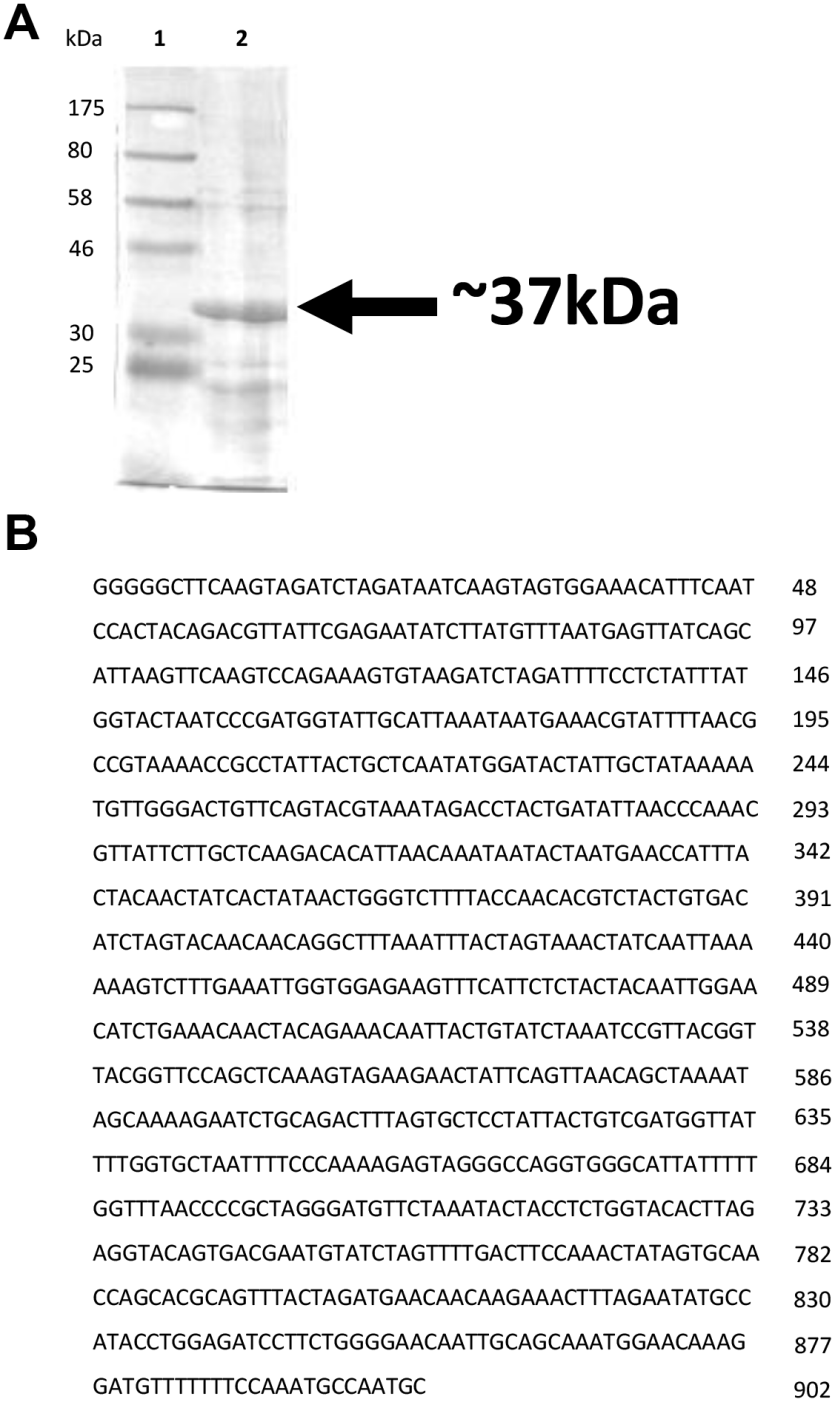
SDS-PAGE analysis of the 37kDa crystal protein of *Bacillus thuringiensis* 4R2 after Coomassie blue staining. (A) Lane 1: Molecular weight marker; lane 2: solubilized pro-PS2Aa1. (B) Nucleotides sequence of the *cry*46Aa1 gene fragment obtained following amplification with the Bt4R2-2F and Bt4R2-1R primer pair.

SDS-PAGE analysis of the solubilized crystal proteins revealed a major band estimated at 37 kDa, corresponding to the native form of the Cry46Aa1 protein (Fig 1). In PCR experiments with Cry46Aa1 specific primers, a 940bp amplification product was obtained. Analyses of the nucleotide sequence using BLAST tools revealed that the nucleotide sequence was 100% homologous with the proteinase K activated Cry46Aa1 nucleotide sequence (NCBI accession number AB099515.1). Owing to this nucleotide sequence identity, the 37 kDa crystal proteins from *B*. *thuringiensis* 4R2 was named PS2Aa1.

### Cytotoxicity of PS2Aa1 in cancer and normal cells

**Fig 2:**
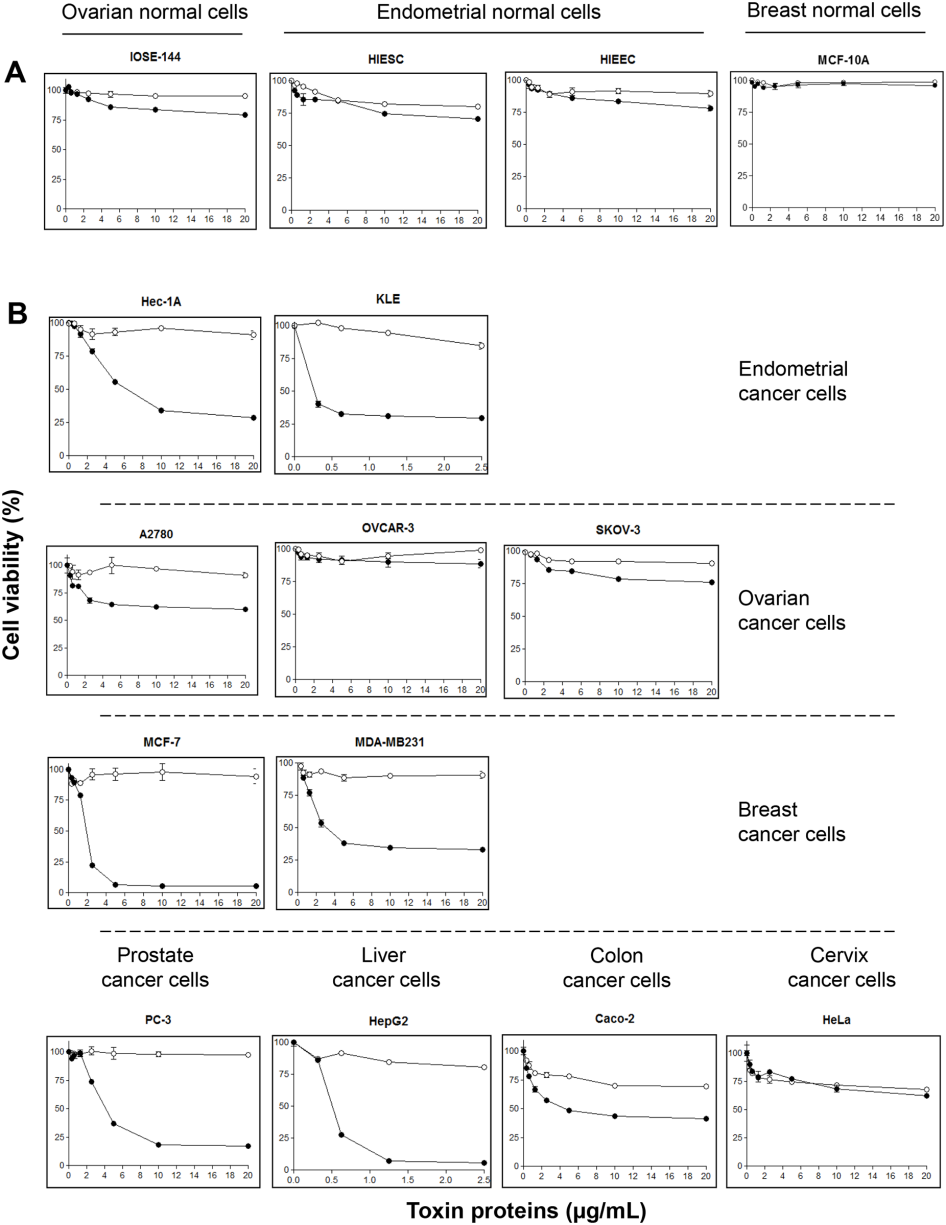
Cytocidal activities of *Bacillus thuringiensis* 4R2 toxin proteins. Normal cultured human cells (A) and cancer cultured human cells (B) (2 X 10^4^ cells) were preincubated at 37°C for 20h; the toxin treated with trypsin (○) or proteinase K (●) was added (final concentrations, 0.3μg/mL to 20μg/mL) further incubated for 24h. Cell proliferation was assayed using MTT.

Trypsin (used as control treatment) and proteinase K activated crystal proteins from *B*. *thuringiensis* 4R2 (PS2Aa1) were examined for cytotoxicity against normal and cancer human cells using MTT Assay for a 24 h treatment (Fig 2). No cytocidal activity was observed after trypsin treatment of solubilized crystal proteins for any of the cells lines. Among the cells tested, proteinase K activated crystal proteins were highly cytotoxic to HepG2, MCF-7, KLE, Hec-1A, MDA-MB231 and PC-3 cells while being moderately cytotoxic to Caco-2 cells. No significant cytotoxic activity was observed in A2780, OVCAR-3, SKOV-3 and HeLa cancer cell lines. None of the normal cell lines (IOSE-144, HIEEC, HIESC and MCF-10A) showed cytotoxic effects. The cytotoxicity of the crystal proteins was dose-dependent and was examined using serial proteins dilutions. Based on their high sensitivity to PS2Aa1, HepG2, PC-3 and MCF-7 cancer cell lines were selected for further experimentations. HIESC cells were used as a normal cell line model to further explore the effects of PS2Aa1 on non-cancer cells.

### Morphological changes in cancer cells induced by PS2Aa1

**Fig 3:**
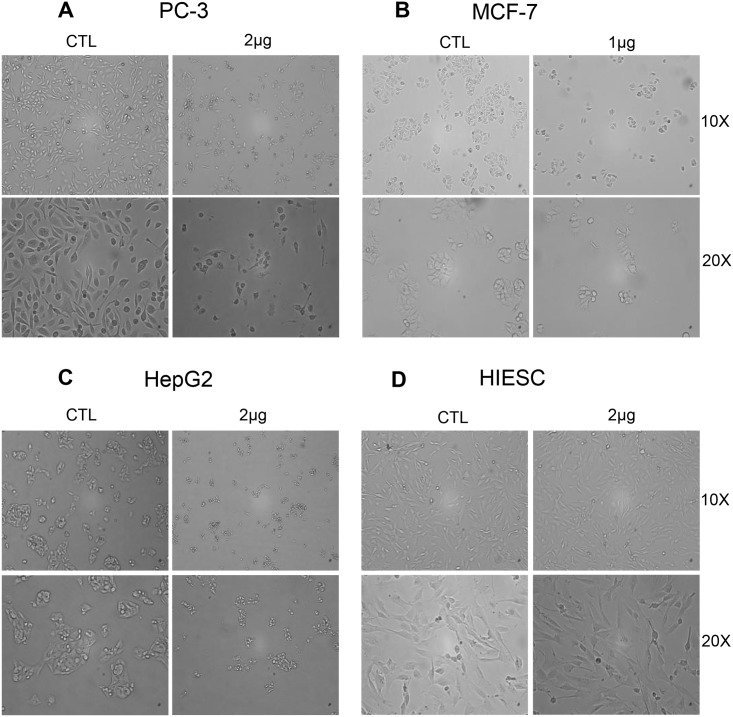
Morphological characteristics of cells following *Bacillus thuringiensis* 4R2 toxin proteins treatment. PC-3 (A), MCF-7 (B), HepG2 (C) and HIESC (D) cells were treated with 1μg/mL or 2μg/mL *B*. *thuringiensis* 4R2 toxin proteins for 24h. After 24h, the cells were observed by light microscopy at a magnification of 10X and 20X. Results shown are representative of three independent experiments.

To further study the effect of PS2Aa1, concentrations from 1μg/mL to 2μg/mL were used based on the previous cell viability experiment. Following treatment by PS2Aa1 proteinase K activated proteins in HepG2 (2μg/mL), PC-3 (2μg/mL) and MCF-7 (1μg/mL) cells, morphological characteristics of the treated cells were observed under light microscopy (Fig 3). Cell shrinkage, characteristic of apoptotic cell death [18], was observed only in HepG2, MCF-7 and PC-3 cancer cells. However, no morphological changes were observed on HIESC; confirming the non-cytotoxicity of PS2Aa1 on normal cells as previously observed with the MTT experiment. No necrosis morphological changes like cell swelling or blebbing were observed [18].

### PS2Aa1 induces cell death by apoptotic mechanisms

**Fig 4:**
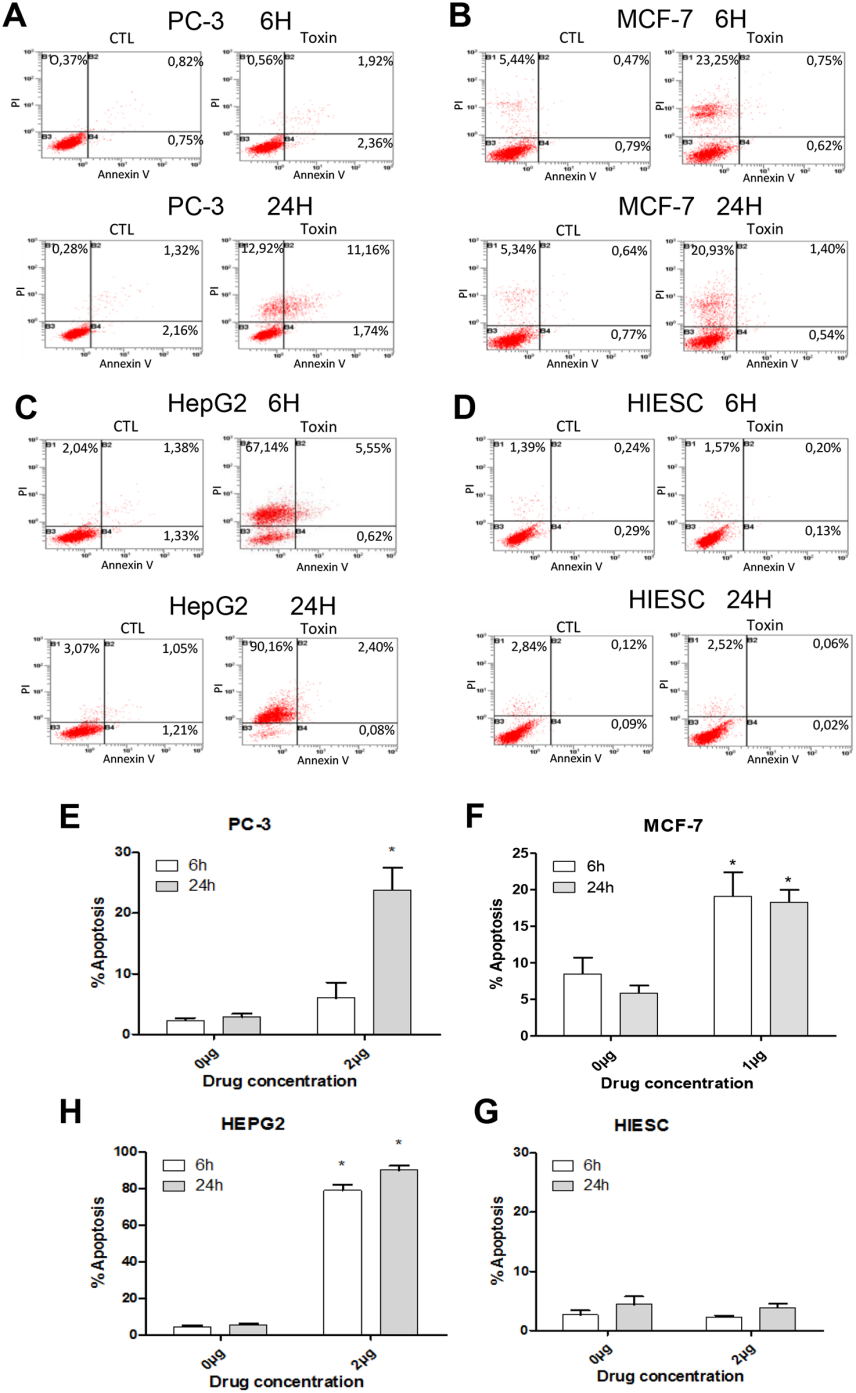
*Bacillus thuringiensis* 4R2 toxin proteins induce apoptosis in human cancer cells. PC-3 (A), MCF-7 (B), HepG2 (C) and HIESC (D) cells were treated with *B*. *thuringiensis* 4R2 toxin proteins (1μg/mL (B) or 2μg/mL (A,C,D)) for 24 h. Annexin V and PI staining was detected by FACS analysis. Results are mean ± S.E.M. of three independent experiments. *P<0.05 compared with corresponding mock-treated cells.

To confirm our previous observations concerning the morphological changes related to apoptosis occurring in cancer cells, we performed annexin V/ propidium iodide (PI) staining analysis on the treated cells. Annexin V has the ability to stain phosphatidyl serine on the outer leaflet of the plasma membrane and its presence on the outer leaflet instead of the inner leaflet is a unique characteristic of apoptosis [19]. Results showed that 6h and 24h after treatment, PS2Aa1 induced high level of apoptosis in all three cancer cell lines (Fig 4A–4C) and very little in the normal endometrial cancer cell line HIESC (Fig 4D) This is in direct correlation with the MTT assay results (Fig 2). Most cancer cells showed high level of apoptosis after 6h of treatment indicating the high efficiency of PS2Aa1 in inducing apoptosis.

**Fig 5:**
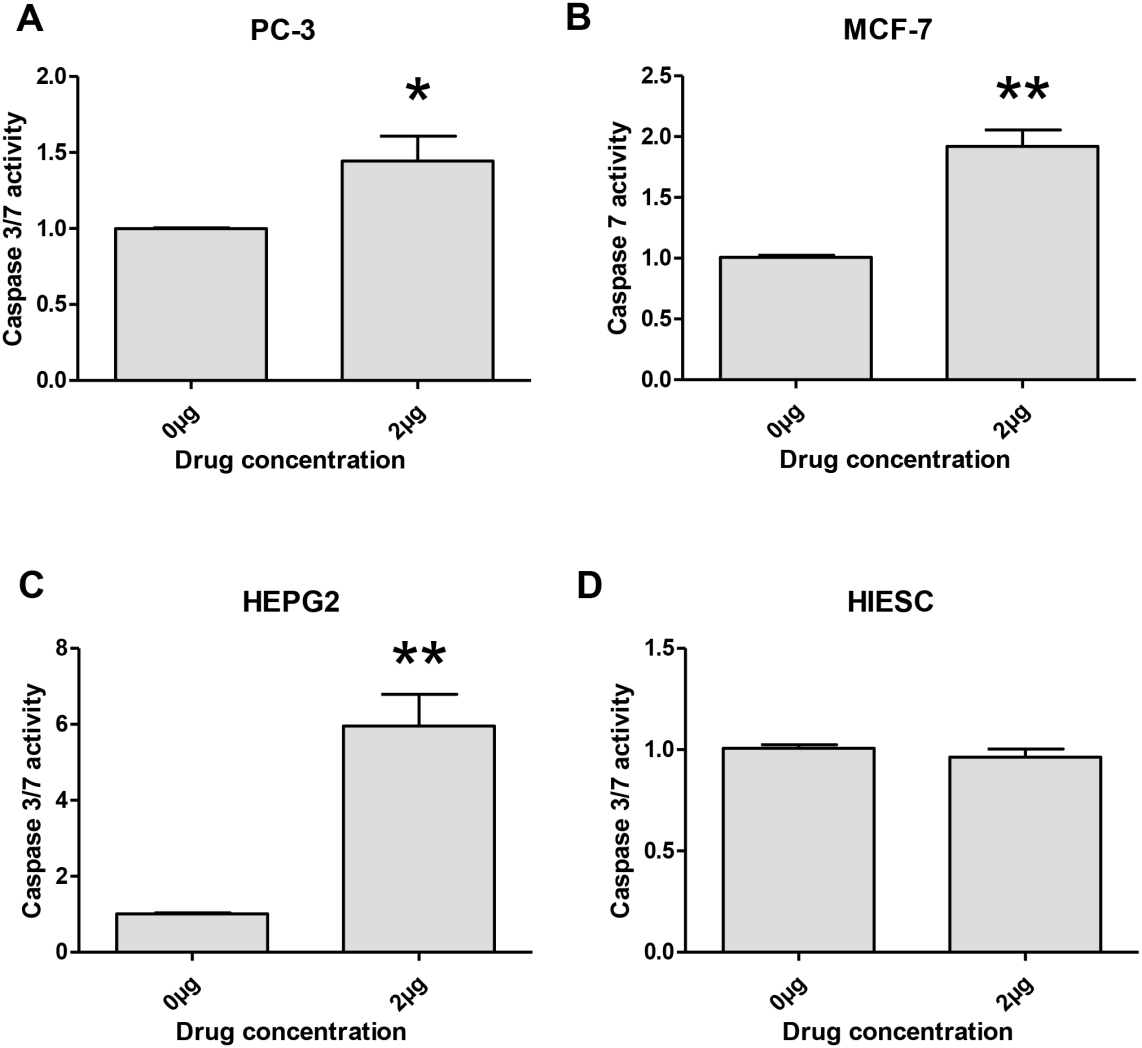
*Bacillus thuringiensis* 4R2 toxin proteins activate and increase caspases-3/7 activity in human cancer cells. PC-3 (A), MCF-7 (B), HepG2 (C) and HIESC (D) cells were treated with *B*. *thuringiensis* 4R2 toxin proteins (2μg/mL) for 24 h. The level of caspases-3/7 was measured using Caspase-Glo 3/7 assay. The MCF-7 (B) cells are Caspase-3 deficient and only Caspase-7 can be measured. *P<0.05 and **P<0.01 compared with corresponding mock-treated cells.

Because PS2Aa1 induces high toxicity and lot of cells are PI positive only after 24h, we decided to perform a caspase-3/7 assay to check if PS2Aa1 specifically activates caspases -3 and -7 to induce apoptosis (Fig 5). After 24h treatment, PS2Aa1 increased significantly the activity of both caspases -3 and -7 in PC-3 and HEPG2 cancer cell lines (Fig 5A and 5C). MCF-7 cancer cell line is caspase-3 deficient and considering this deficiency, only caspase-7 can be measured by this assay in this cell line [20]. A significant increase of caspase activity can be observed in MCF-7 cancer cell line which indicates that the apoptosis induced is compensated by caspase-7, implicated in the mechanism of action of PS2Aa1 (Fig 5B). Low level of cell death was previously observed in normal cell line HIESC by Annexin V/PI flow cytometry and consequently, no significant increase of caspase-3/7 activity could be measured by the caspase assay in HIESC (Fig 5D).

**Fig 6:**
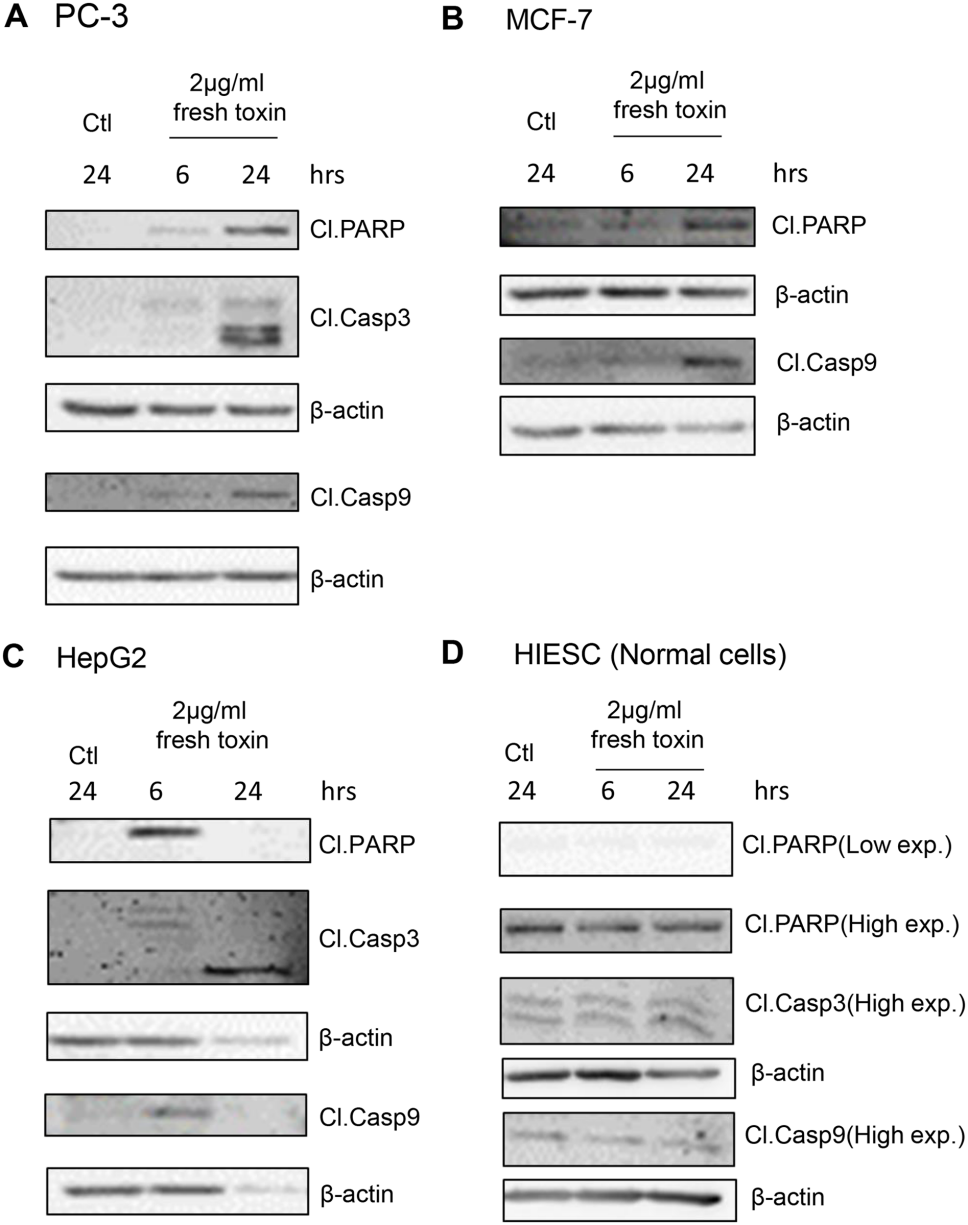
*Bacillus thuringiensis* 4R2 toxin proteins activate apoptosis mechanisms in human cancer cells. PC-3 (A), MCF-7 (B), HepG2 (C) and HIESC (D) cells were treated with *B*. *thuringiensis* 4R2 toxin proteins (1–2μg/mL) for 6h and 24 h. The levels of apoptosis–specific cleaved proteins Caspase-3, Caspase-9 and PARP were determined in treated cells using western blot analysis. The MCF-7 (B) cells are Caspase-3 deficient. β-Actin was used as a loading control. Results shown are representative of three independent experiments.

To further investigate apoptosis induction, we also measured different apoptosis markers by western blot analysis such as cleaved caspase-3, 8, -9 and cleaved PARP. Our results showed that, as early as 6h of treatment with PS2Aa1, caspase-3 cleavage/activation was observed in both PC-3 and HEPG2 cancer cells (Fig 6A and 6C; MCF-7 being caspase-3 negative). Once activated, caspase-3 is an effector caspase and can do proteolytic cleavage on various proteins, such as the repair protein PARP, then leading to apoptotic cell death. However, caspase-3 requires initiator caspases from either the intrinsic or extrinsic pathway to be cleaved/activated [21,22]. By western blot analysis, we measured cleaved caspase-8 (extrinsic pathway) and cleaved caspase-9 (intrinsic pathway) and found out that only caspase-9 cleavage/activation was present in all three cancer cell lines (Fig 6A–6C) while caspase-8 cleavage/activation was not observed (Data not shown). In correlation with caspases cleavage, PS2Aa1 also induced PARP cleavage/degradation in all three cancer cell lines (Fig 6A–6C). Cleaved PARP and cleaved caspase-3 protein levels are low after 24h treatment of the toxin in HEPG2 cancer cell line (Fig 6C). This is probably because most of the cells were dead after 6h (>75%). After 24h of treatment, almost all the HEPG2 cells were floating and dead (>92%). Proteins were probably all degraded because of proteases action in late apoptosis leading to massive protein and cellular destruction [23]. This is further supported by the MTT data (Fig 2B) and Annexin V/PI flow cytometry (Fig 4C and 4H) which indicate that almost 100% of the cells are dead at this concentration after 24h of treatment explaining the situation observed. It is also notable that none of these markers of apoptosis were observed in the normal cell line HIESC (Fig 6D) indicating that no apoptosis was occurring using the maximum dose of PS2Aa1 (2μg/ml) previously used on cancer cell lines. The Western blot bands visible in the normal cell line HIESC are the basal level of the different markers, observable only after high exposition to reveal the appropriate protein bands (Fig 6D). This is in concordance with the absence of apoptosis and caspases-3/7 activity observed in previous experiments for the normal cell line HIESC (Figs 4 and 5).

### Regulation of different survival and death pathways in response to PS2Aa1

**Fig 7:**
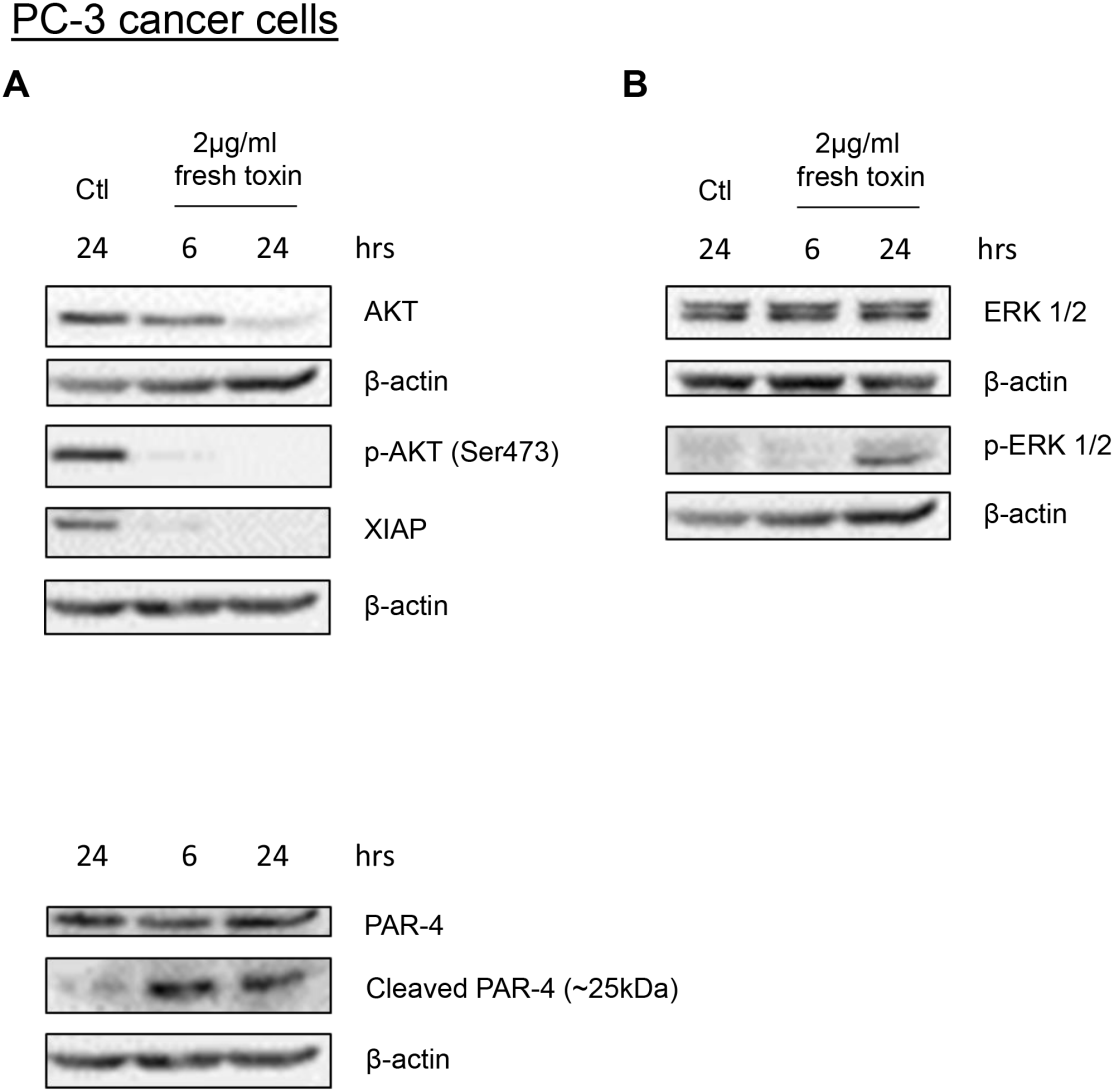
*Bacillus thuringiensis* 4R2 toxin proteins regulate various survival pathways in PC-3 cancer cells. PC-3 cancer cells were treated with Bt 4R2 toxin proteins (2μg/mL) for 6h and 24 h. (A) PI3K/AKT pathway proteins (B) ERK and (C) PAR-4 protein levels were determined in treated cells using western blot analysis. β-actin was used as a loading control. Results shown are representative of three independent experiments.

All the previous experiments demonstrate that PS2Aa1 toxin can induce cell death in cancer cells through apoptosis induction. In order to investigate the possible involvement of other survival/death pathways, we performed additional experiments on PC3 prostate cancer cells using western blot analysis. We first investigated the AKT survival pathway known for being an important survival mechanism for cancer cells. Proteins from the AKT pathway are often altered in cancer and the high survival signal from these mutations are often responsible for the resistance of cancer cells to therapeutics treatments and the impossibility to induce apoptosis [24–28]. Our results showed a high decrease of phosphorylated (the active form) AKT at serine 473. Total AKT level was also decreased suggesting that it is also in part responsible for the decrease of p-AKT level. XIAP, an inhibitor of caspase and a ubiquitin ligase for PTEN (a negative regulator of AKT phosphorylation) was also decreased (Fig 7A) [29]. ERK1/2, proteins of the MAPK pathway, requires activation/phosphorylation to induce apoptosis in cancer cells following treatment with cisplatin or other apoptosis stimuli [30–32]. As observed in cisplatin treatments, PS2Aa1 induced an increase of ERK1/2 phosphorylation at 24h following treatment while total ERK level was staying stable (Fig 7B). This suggests that ERK activation is required for the induction of apoptosis. Finally, we recently discovered a new mechanism related to the tumour suppressor PAR-4 which is cleaved by caspase-3 upon apoptosis stimuli and is able to induce apoptosis once cleaved [33,34]. Because of PS2Aa1 capacity to activate apoptosis mechanisms, we questioned whether PAR-4 cleavage could be involved in apoptosis induction upon treatment with the toxin. Interestingly, a cleaved fragment of approximately 25 kDa appeared as soon as 6h after treatment with PS2Aa1 in full correlation with our hypothesis (Fig 7C). The presence of the cleaved PAR-4 fragment, normally present only in cancer cells undergoing apoptosis, reinforces the idea that PS2Aa1 is an inducer of apoptosis in cancer cells.

### Inhibition of MAPK and Pi3K survival pathways in combination with PS2Aa1

**Fig 8:**
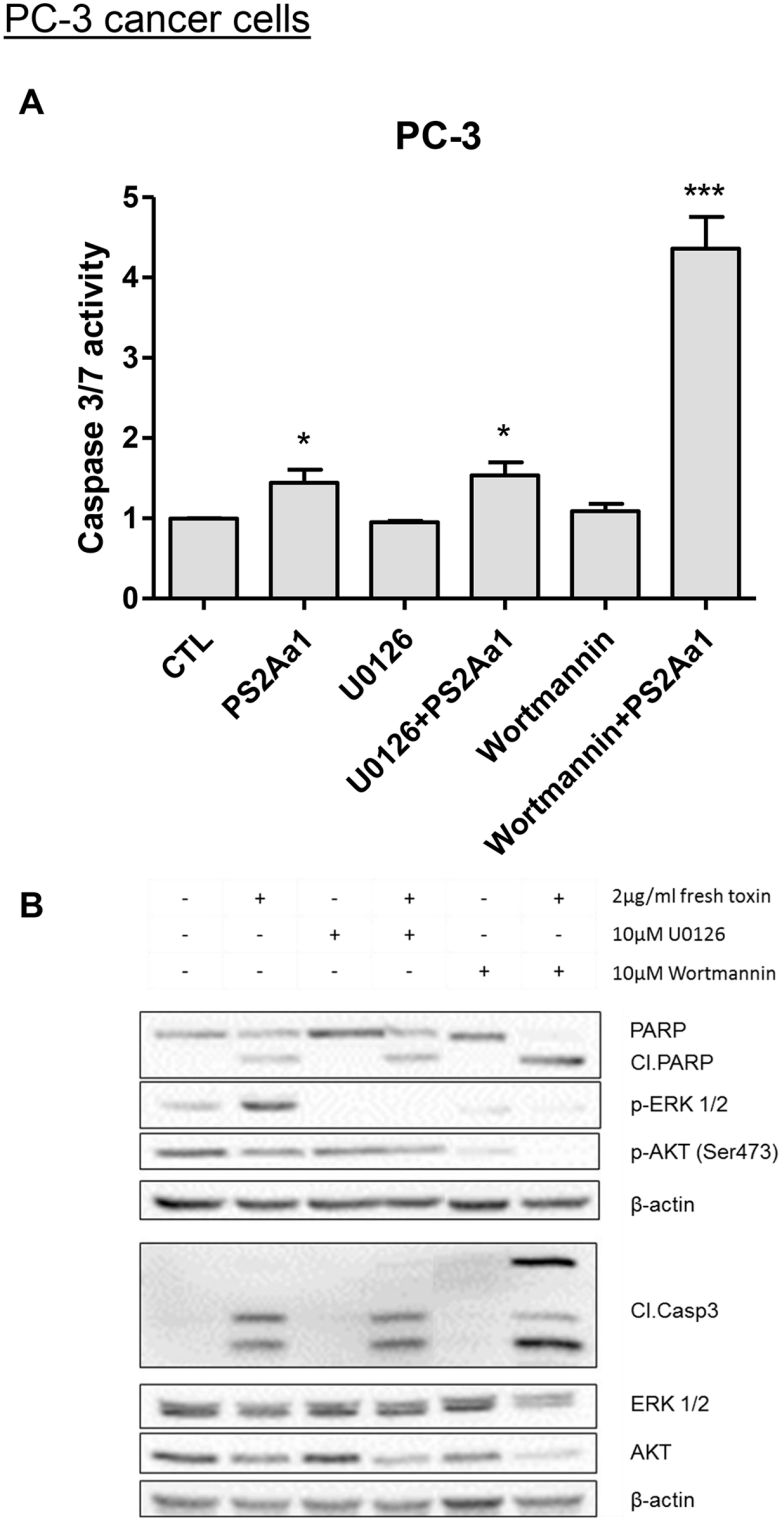
*Bacillus thuringiensis* 4R2 toxin proteins in combination with MAPK and PI3k survival pathways inhibitors. PC-3 cancer cells were pre-treated with MAPK inhibitor U0126 (10μM) or PI3k inhibitor Wortmannin (10μM) for 1 h and then Bt 4R2 toxin proteins (2μg/mL) was added for 24h. (A) The level of caspases-3/7 was measured using Caspase-Glo 3/7 assay. *P<0.05 and ***P<0.001 compared with corresponding mock-treated cells. (B) The levels of AKT (total and phosphorylated), ERK1/2 (total and phosphorylated), cleaved Caspase-3 and cleaved PARP were determined in treated cells using western blot analysis. β-actin was used as a loading control. Results shown are representative of three independent experiments.

Considering the previous results obtained, indicating that the toxin PS2Aa1 regulates positively ERK1/2 phosphorylation (MAPK pathway) and negatively AKT phosphorylation at serine 473 (AKT pathway), we decided to use inhibitors for both pathways in combination with PS2Aa1 to see if these pathways influence apoptosisinduction and caspases activity in PC3 cancer cell line. We used U0126 as an inhibitor of the MAPK pathway (ERK1/2) and wortmannin as an inhibitor of the Pi3K pathway (AKT), alone or in combination with PS2Aa1. To see the impact of the inhibitors in combination with PS2Aa1 in PC3 cancer cells, we performed a caspase 3/7 assay (Fig 8). Interestingly, when using Pi3K inhibitor wortmannin in combination with PS2Aa1, we observed a synergetic increase of caspase 3/7 activity (Fig 8A). This observation is also correlated with the western blots where an increase of cleaved caspase-3 protein level was observed (Fig 8B). Indeed, in relation with the observed increase of active caspase-3, PS2Aa1 in combination with wortmannin also increased the level of PARP cleavage/degradation and almost a complete reduction of full length PARP Fig 8B). No significant changes were observed in both experiments when using the MAPK inhibitor U0126 in combination with PS2Aa1. ERK1/2 and AKT protein levels (total and phosphorylated) were measured to confirm the efficiency of both inhibitors after 24h treatment.

## Discussion

Parasporin-2Aa1 is a 37kDa crystal protein, originally isolated from *B*. *thuringiensis* A1547, known to be a pore forming toxin active against human cancer cells [35]. The previous studies suggest that this toxin protein mode of action is non-apoptotic but these studies lack in-depth molecular analysis of cancer cell death pathways [11,12]. In the present study, a new *B*. *thuringiensis* strain, 4R2, was shown to produce the PS2Aa1 (Cry46Aa1) crystal protein. We first observed that the proteinase K treated PS2Aa1 crystal proteins caused morphological changes in the highly sensitive HepG2, MCF-7 and PC-3 cancer cell lines while, on the other hand, the normal cell line HIESC morphology was unchanged. In previous studies, cell lysis, swelling and blebbing were observed in sensitive cell lines including HepG2 [11,12]. In this study, none of these morphological changes were observed; instead, cells shrinkage was observed in the sensitive cells lines, supporting the hypothesis that PS2Aa1 could induce apoptosis in cancer cells line [18].

The *B*. *thuringiensis cry* genes encoding the Cry proteins are located on plasmids. The latter can be transferred between *B*. *thuringiensis* strains [2]. Such plasmid transfers may explain why the gene encoding the protein PS2Aa1 (Cry46Aa1) can be found in two different *B*. *Thuringiensis* strains. As shown in a previous study, *cry* genes coding for the same parasporin protein can be geographically dispersed [36]. This supports the fact that the *B*. *thuringiensis* 4R2 strain contains the same parasporin gene as *B*. *thuringiensis* A1547. The 1017 nucleotide sequence (338 amino acids) of *cry46Aa1* from *B*. *thuringiensis* A1547 strain (Genbank accession number: AB099515.1) encodes the full length Cry46Aa1 protoxin, the precursor of the activated toxin. When activated by proteinase K, the protoxin is cleaved at residues 52 and 306 of the amino acids sequence to yield an active form [11]. The nucleotide sequence from *B*. *thuringiensis* 4R2 obtained with our primers by PCR amplification is 100% homologous with the sequence of proteinase K activated protein described above (amino acids residues 52 to 306). The Cry46Aa1 protein expression was confirmed by SDS-PAGE analysis and the 37kDa major band observed indicates that the crystal proteins solubilisation was efficient.

Dose-response studies of proteinase K and trypsin activated PS2Aa1 crystal protein were conducted using the MTT assay. When activated by proteinase K, PS2Aa1 had high cytotoxicity against HepG2 cells as previously reported [12]. The normal cell lines HIESC, IOSE-144, HIEEC and MCF-10A were not sensitive to the proteinase K activated proteins PS2Aa1. Moreover, several new cancer cells lines were screened and new sensitive cancer cell lines, MCF-7, MDA-MB231, KLE, Hec-1A and PC-3, were found. In susceptible insect models, activated Cry proteins bind to specific receptors at the surface of the epithelial membrane midgut cells [37]. In many case, Cry proteins bind to multiple receptors sequentially leading to pore formation in the midgut cells [38]. The selective cytotoxicity of PS2Aa1 toward cancer cells and the absence of cytotoxicity in the normal cells suggest that either different receptors are present on the surface of cancer and normal cell membranes or only absent on the normal cells. Alternatively, normal cells may not express GPI anchor protein to enhance cytotoxicity of PS2Aa1.

When activated by trypsin, the PS2Aa1 crystal protein did not show cytotoxicity against the different cell lines. PS2Aa1 protoxin had to be cleaved by proteinase K to be cytotoxic against human cancer cells [12]. Presumably, the trypsin and proteinase K cleavage sites are different. Toxin proteins without protease activation were tested at high concentration (20μg/mL) against all cells lines and no toxicity was observed in all the cells treated (data not shown). In insect models, specific protease activation is essential and can determine the toxin specificity [39]. Digestion of Cry proteins with some proteases can also generate inactive products and contribute to insect resistance [40]. It is reasonable to hypothesize that without proper protease activation, the PS2Aa1 protoxin could not be recognized by the normal cells receptors. Conversely, when cleaved with proteinase K, specific regions of the activated PS2Aa1 can bind to a receptor. When cleaved by trypsin, theses specific regions can only be partially exposed or not at all, preventing binding.

The parasporins are not the only bacterial toxins able to induce apoptosis in host cells [41]. Mitochondria pathway apoptosis can also be activated by bacterial pathogens [42]. PS2Aa1 shows structural homology with the *Aeromonas hydrophila* aerolysin and *Clostridium perfringens* alpha-toxin [35]. These pore forming toxins require GPI-anchored proteins for efficient cytolysis and binding [43]. These structural similarities strengthen the indications that PS2Aa1 is a pore forming protein [35]. In the PS2Aa1, the GPI-anchored proteins seem to be implicated in toxin cytotoxicity but not in binding specificity as opposed to aerolysin and alpha-toxin [13]. The lack of information on the binding events is a hurdle in gaining a better understanding of the molecular pathways involved at this level. One hypothesis is that PS2Aa1 forms small pores in the plasma membrane enabling the passage of ion molecules in cells and thus inducing apoptosis. This is the mode of action displayed by the *Staphylococcus aureus* alpha toxin which also induces apoptosis in host cells [44].

So far, some other parasporins have been shown to induce apoptosis in mammalian cancer cells [45,46]. However, we are the first to demonstrate the apoptotic effects of parasporin-2Aa1 (PS2Aa1). The parasporin-1Aa1 is cytotoxic against HeLa and MOLT-4 cells and cause an increase of intracellular level of calcium via heterotrimeric G-protein or G-protein-coupled receptors [46]. This parasporin, however, is not considered a pore forming toxin, contrary to PS2Aa1. Another 29 kDa parasporin from the *B*. *thuringiensis* A1519 strain induces mitochondrial apoptosis pathway in the Jurkat cells via caspase-3 and -9 cleavage and the release of cytochrome c from the mitochondria [45]. In a previous study, it was reported that HepG2 cells, treated with PS2Aa1, have shown a leakage of cytochrome C from mitochondria who was not consider at that time as an apoptotic event [11]. The cytochrome C leakage from the fragmented mitochondria is hallmark of apoptosis induction through the mitochondria pathway [47]. In our current study, caspase-9 cleavage is observed in the three PS2Aa1 selected sensitive cells lines (HepG2, MCF-7 and PC-3) supporting the hypothesis of the mitochondria pathway activation. Identification of the receptor implicated in PS2Aa1 binding will contribute to a better understanding of the exact molecular events involved in apoptosis induced cell death.

The regulation of the various survival pathway observed supports the hypothesis of the induction of apoptosis by PS2Aa1 (Fig 6). AKT pathway is well known for its survival and proliferative functions [24,48]. Survival aspect of the AKT pathway involves the inactivation of proapoptotic factors such as Bad and caspase-9 but also the promotion of the cell cycle via the activation of mTor/p70S6k cascade [24,29]. XIAP is a protein responsible for being an inhibitor of caspase-3, -7 and- 9 by directly binding these proteins and then inhibiting their functions [49]. By inhibiting caspase-9, XIAP can also interfere with cytochrome C pathway. It has been shown that XIAP anti-apoptotic functions are mediated in part by the regulation of the AKT pathway [29]. Upon apoptosis stimuli, AKT, its active form p-AKT and XIAP are very often downregulated to block proliferation and enhance apoptotic mechanisms as observed upon treatment with PS2Aa1 on various cancer cells [28,50]. This is further supported by the cleavage/activation of caspase-3, -7and -9. Currently, in cancer clinical trials, many PI3k inhibitors are under study and some of these are very promising. Using pathway inhibitors in combination with conventional chemotherapy is often very valuable by increasing the efficiency of the treatment and also counter chemoresistance by decreasing the activity of these survival pathways [51–53]. Following this idea of drug combination, we observed for the first time the synergetic effect of PS2Aa1 toxin in combination with Wortmannin (PI3k inhibitor). Indeed, by treating prostate cancer cells PC-3 with this combination, apoptosis was increased by more than three time when compared with the toxin alone. This synergetic effect of PS2Aa1 with Wortmannin would probably be even higher in PTEN mutant cancer, very frequent in the endometrium [54]. This is valuable information for future experiments and pre-clinical trial involving the PS2Aa1 toxin considering the arrival of many PI3k inhibitors in clinical and the more common use of combination therapy for the treatment of cancer patient.

ERK1/2 are proteins from the MAPK pathway and known for various regulation such as proliferation, cell migration, differentiation and cell death depending on the condition exposed [31,32]. Cell death has been frequently associated with p-ERK1/2 upon diverse apoptotic stimuli [30–32]. The reason why p-ERK1/2 is increased and implicated when apoptosis occurs is not well known. However, some papers indicates that P53 activity can be upregulated by the ERK1/2 during apoptosis. Also, it has been shown that using MEK inhibitors reduce apoptosis and decreases the level of many apoptotic factors such as TNF-α and cleaved-Caspase-3 [32,55–57]. Similar results were obtained upon treatment with PS2Aa1 (Fig 6) with the increase of the active form of ERK1/2 (p-ERK1/2) support the implication of the MAPK pathway in enhancing apoptosis activity as observed with cisplatin and other apoptotic inducers [30–32]. However, when using a MAPK inhibitor, U0126, in combination with PS2Aa1, no change in apoptosis were observed when compared with the toxin alone. This indicates that the MAPK is not critical for the mechanism of action of the toxin.

Prostate apoptosis response-4 (PAR-4) is a tumour suppressor known for inducing selectively apoptosis in cancer cells using a unique domain (SAC) [58]. PAR-4 can induce apoptosis by both the intrinsic and extrinsic pathways involving various regulation of these different pathways. However, the selectivity of PAR-4 to induce apoptosis only in cancer cells is attributed to the PKA level required to activate PAR-4 which is not sufficient in normal cells compared to most cancer cells [59,60]. More recently, we discovered that PAR-4 is cleaved by caspase-3 upon apoptotic stimuli and that its cleaved fragment plays a role in the apoptotic mechanism of PAR-4 in cancer cells [33]. Upon treatment with PS2Aa1, a cleaved fragment of PAR-4 was observed in the PC-3 cancer cell line further supporting the hypothesis of the induction of apoptosis by the toxin and its selectivity for cancer cells.

In conclusion, the results obtained in this study clearly indicate that PS2Aa1, synthesized by *B*. *thuringiensis* 4R2, induces apoptosis in human cancer cells but not in normal cells. Caspase activation and the regulation of the tested survival/death pathways involved in apoptosis confirm that the PS2Aa1 induces cell death by apoptosis and can now better explain PS2Aa1 mechanism of action. The PS2Aa1 toxin is a very promising molecule for future studies on its selectivity on cancer cells. However, further experiments on the mechanism implicated in cell specificity and the mechanism related to the induction of apoptosis will be needed in order to clarify its mode of action.
